# Importance of Comprehensive Molecular Profiling for Clinical Outcome in Children With Recurrent Cancer

**DOI:** 10.3389/fped.2018.00114

**Published:** 2018-04-20

**Authors:** Olga Østrup, Karsten Nysom, David Scheie, Ane Y. Schmidt, Rene Mathiasen, Lisa L. Hjalgrim, Tina E. Olsen, Jane Skjøth-Rasmussen, Birthe M. Henriksen, Finn C. Nielsen, Peder S. Wehner, Henrik Schrøder, Astrid M. Sehested, Catherine Rechnitzer, Maria Rossing

**Affiliations:** ^1^Center for Genomic Medicine, Copenhagen University Hospital, Copenhagen, Denmark; ^2^Department of Pediatrics and Adolescent Medicine, Copenhagen University Hospital, Copenhagen, Denmark; ^3^Department of Pathology, Copenhagen University Hospital, Copenhagen, Denmark; ^4^Department of Neurosurgery, Copenhagen University Hospital, Copenhagen, Denmark; ^5^Department of Radiology, Rigshospitalet, Copenhagen University Hospital, Copenhagen, Denmark; ^6^HCA Hospital for Children, University of Southern Denmark, Odense University Hospital, Odense, Denmark; ^7^Department of Clinical Medicine – Department of Pediatrics, Aarhus University Hospital, Aarhus, Denmark

**Keywords:** recurrent cancer, children, molecular profiling, precision medicine, clinical intervention

## Abstract

**Purpose:** Pediatric cancers are often difficult to classify and can be complex to treat. To ensure precise diagnostics and identify relevant treatment targets, we implemented comprehensive molecular profiling of consecutive pediatric patients with cancer relapse. We evaluated the clinical impact of extensive molecular profiling by assessing the frequency of identified biological onco-drivers, altered diagnosis, and/or identification of new relevant targeted therapies.

**Patients and Methods:** Forty-six tumor samples (44 fresh-frozen; two formalin-fixed paraffin embedded), two bone marrow aspirates, three cerebrospinal fluid samples, and one archived DNA were obtained from 48 children (0–17 years; median 9.5) with relapsed or refractory cancer, where the disease was rapidly progressing in spite of their current treatment or they had exhausted all treatment options. The samples were analyzed by whole-exome sequencing (WES), RNA sequencing (RNAseq), transcriptome arrays, and SNP arrays. Final reports were available within 3–4 weeks after patient inclusion and included mutation status, a description of copy number alterations, differentially expressed genes, and gene fusions, as well as suggestions for targeted treatment.

**Results:** Of the 48 patients, 33 had actionable findings. The most efficient method for the identification of actionable findings was WES (39%), followed by SNP array (37%). Of note, gene fusions were identified by RNAseq in 21% of the samples. Eleven findings led to clinical intervention, i.e., oncogenetic counseling, targeted treatment, and treatment based on changed diagnosis. Four patients received compassionate use targeted therapy. Six patients experienced direct benefits in the form of stable disease or response.

**Conclusion:** The application of comprehensive genetic diagnostics in children with recurrent cancers allowed for discovery and implementation of effective targeted therapies and hereby improvement of outcome in some patients.

## Introduction

**Figure 1:**
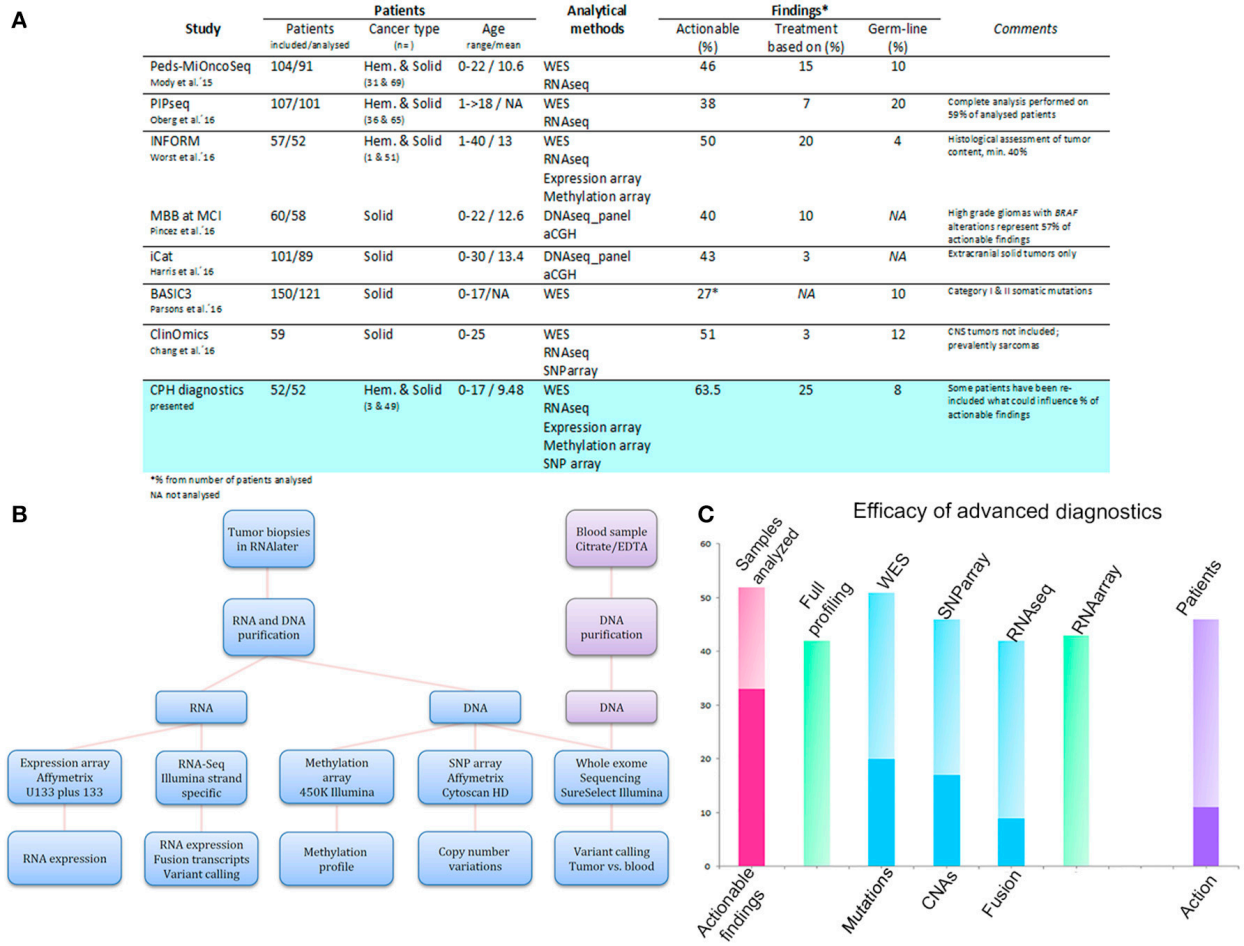
Comparison of the genetic diagnostics presented here with those reported in other studies **(A)**, analytical pipeline of comprehensive molecular profiling for pediatric cancer patients **(B)**, and efficacy of advanced diagnostics **(C)**. **(A)** Table summarizing the findings of our review and those of other studies for a performance comparison. **(B)** Scheme illustrating the analytical set-up for high-throughput profiling of recurrent pediatric cancers at the Center for Genomic Medicine, Copenhagen University Hospital. **(C)** Graph illustrating the efficacy of advanced diagnostics in the initial period after their implementation. The y-axis indicates the number of samples analyzed in total (pink column), analyzed by all the methods (green column to the right), and analyzed by the individual methods (blue columns), respectively. The deep pink segment in the first column denotes the number of samples with an actionable finding. The deep blue segments for the individual methods delineate the number of samples with a positive finding, i.e., mutations for WES, CNAs for SNP arrays, and gene fusions for RNAseq. The last column indicates the number of included patients (*n* = 46) and the deep violet segment denotes the number of patients where clinical action was undertaken based on the findings.

Precision medicine and targeted treatment have been the main focus of adult oncology for more than a decade. In striking contrast, only a few recent studies have reported the efficacy and importance of precision medicine in pediatric cancers (for references see Figure 1A). Moreover, whereas research in adult oncology mostly focuses on the discovery and testing of potential drugs and targets (actionable targets), a considerably large part of pediatric oncology research is still dedicated to understanding the etiology of cancer. Therefore, molecular findings influencing any kind of clinical intervention for pediatric patients (actionable findings) are of a great importance for development in the field.

In pediatric oncology, several factors hinder the process of discovering new efficient treatments. First, pediatric cancers are rare [1] and their study requires efficient international consortia. Secondly, pediatric cancers generally contain far fewer genomic alterations [mutations, copy number alterations (CNAs)] than adult cancers, thus significantly restricting the use of targeted treatment. Ironically, in many instance, targeted therapies in children rely on the presence of adult-type accessory targets by chance present in the tumor and rarely being the driver alteration specific to the children. Many driver mutations of pediatric cancer do not have a targeted therapy available. Thirdly, most childhood cancers lack frequently shared genetic alterations [2, 3]; in addition, this makes the classification of tumors more challenging [4]. Consequently, pediatric tumors have a high degree of interpersonal heterogeneity, returning precision medicine back into the era of risk-stratified treatment strategies. This situation may therefore explain why the majority of pediatric solid cancers still await a major breakthrough in the exploration of targeted treatment.

Hence, in order to select an efficient treatment, each patient has to be considered as a unique case, with personalized diagnostics being the main focus of precision medicine in pediatrics. In line with this, several recent studies in pediatric oncology were based on providing a complex view of the disease [5–10]. Specifically, the tumors of included patients were profiled in order to gather information about the molecular aberrations causing the disease and, in parallel, to identify targets for potential therapeutic agents (Figure 1A).

From a methodological point of view, the studies mostly relied on whole-exome sequencing (WES) for the detection of somatic and germline mutations. Occasionally, WES was also applied for the derivation of CNAs. However, it became clear that the application of several analytical platforms leads to a higher success rate in the identification of actionable findings [10]. Hence, a comprehensive screening design, i.e., including WES, RNA sequencing, and transcriptome, SNP, and DNA methylation arrays, was shown to be the most efficient tool for the description of a patient's disease and determination of possible treatment targets.

The promising results achieved in these studies led to the implementation in 2015 of new diagnostics for refractory and relapsed pediatric cancers comprising high-throughput molecular profiling at the Center for Genomic Medicine, Rigshospitalet, Copenhagen, Denmark. This review reports the results and remarks acquired during the initial 18 months of these new diagnostics.

## Patients and methods

### Patients

From August 2015 until January 2017, pediatric cancer patients with recurrent disease were offered complete genomic profiling of their tumors if all general treatment schemes had been exhausted and the disease continued to progress. The aim of this analysis was to search for potential targets for experimental treatment and/or clarify the diagnosis in light of inconclusive results from standard diagnostic work-outs. Patients were between 0 and 17 years of age and were suffering from recurrent solid or hematological cancers (Table 1; Table S1). Three rare and diagnostically challenging cases were included at earlier stages of the disease with the aim of providing a precise, molecular-based diagnosis. Legal guardians provided written/oral and informed consent and all studies were conducted in accordance with the Declaration of Helsinki. Publication of results was approved by the local ethics committee in the Capital Region of Denmark (H-4-2010-050). A specific written consent was obtained from legal guardians and/or patients presented in the specific cases examples. At the time of inclusion, blood samples were collected in parallel with ultrasound-guided, surgical or stereotactic tumor biopsies. In total, 52 samples from 48 patients were included for molecular profiling (Table 1; Table S1; Supplementary Material).

**Table 1:** Overview of patients' histopathological diagnoses and potential driver genomic alterations/actionable findings.

	**Sample #**	**Age (y)**	**Histopathological diagnosis at inclusion**	**WHO grade**	**Genomic alteration *(Known prior profiling)***
CNS tumors	1	6–10	*Atypical meningioma*	II	TFG-ROS1 fusion
	2	11–15	*Choroid plexus carcinoma/Malignant peripheral nerve sheath tumor*	III	HRD; amplification *MET, CDK6, NOTCH*; loss *RB1*
	3	0–5	*Pilocytic/pilomyxoid astrocytoma*	I-II	NFIA-RAF1 fusion
	4	0–5	*Glioblastoma*	IV	
	5	11–15	*Pleomorphic xanthoastrocytoma*	II-III	*BRAF* V600E; loss *CDKN2A/B*
	8	0–5	*Ganglioglioma / Diffuse astrocytoma*	I-II	
	12	11–15	*Diffuse midline glioma H3K27M-mutated*	IV	H3F3 K28M; *TP53* c.469_471delGTC
	13	0–5	*Anaplastic ependymoma*	III	
	14	6–10	*Juvenile xanthogranuloma*		
	15 (13)	0–5	*Anaplastic ependymoma*	III	
	16	11–15	*Anaplastic pleomorphic xanthoastrocytoma/Glioblastoma*	IV	*BRAF* V600E; *ATRX* R1739^*^; loss *CDKN2A/B*
	17 (8)	0–5	*Ganglioglioma/Diffuse astrocytoma*	I-II	FGFR3-TACC3 fusion
	18	6–10	*Diffuse midline glioma H3K27M-mutated*		H3F3 K28M; *TP53* F134S & c.376-1G>A; *PIK3R1* c.936-2A>T
	21	6–10	*Glioblastoma*	IV	Mismatch repair deficiency
	22 (8)	ND	*Ganglioglioma/Diffuse astrocytoma*	I-II	FGFR3-TACC3 fusion
	23	>16	*Malignant peripheral nerve sheath tumor*		Amplification *FGFR1*
	24	6–10	*Pilocytic astrocytoma*	I	
	25	>16	*Pilocytic/pilomyxoid astrocytoma*	I-II	*FGFR1* N546K; *PTPN11* E69K
	28 (2)	11–15	*Choroid plexus carcinoma/Malignant peripheral nerve sheath tumor*	III	HRD; loss *RB1*; amplification *MET, CDK6, NOTCH2*
	31	0–5	*Anaplastic ependymoma*	III	
	34	>16	*Atypical neurocytoma*	II	
	35	11–15	*Chondroblastic osteosarcoma*		HRD
	36	0–5	*Astrocytoma*		*KRAS* E63K
	37	0–5	*Neuroblastoma*		LOH; amplification *MET, JAG1*
	38	0–5	*Anaplastic ependymoma*	III	
	39	11–15	*Pineoblastoma*	IV	
	41 (39)	11–15	*Pineoblastoma*	IV	
	44	11–15	*Diffuse astrocytoma*	II	MMRD
	46	11–15	*Malignant peripheral nerve sheath tumor*		
	49 (39)	11–15	*Pineoblastoma*	IV	
	50	6–10	*Anaplastic ependymoma*	III	MN1-BEND2 fusion; loss X
	51	>16	*Rhabdomyosarcoma*		PAX3-FOXO1 fusion
Extracranial solid tumors	6	0–5	*Mesoblastic nephroma*		amplification *ERVV1/2*
	7	11–15	*Signet ring cell carcinoma*		
	9	>16	*Chondrosarcoma*		*IDH1* R132L
	10	11–15	*Chordoma, dedifferentiated/anaplastic type (INI1-loss)*		*PIK3CG* G1058R
	11	11–15	*Hepatoblastoma*		*CDKN2A* L78fs^*^41
	19	11–15	*Alveolar rhabdomyosarcoma*		PAX3-FOXO1 fusion
	26 (7)	ND	*Signet ring cell carcinoma; immune therapy screening*		
	27	>16	*Nephroblastoma*		amplification *NGFR*; *TP53* c.75-1G>C; *GNA11* R183C
	29	0–5	*Neuroblastoma*		*ALK* F1174L; amplification *MYCN*
	30	0–5	*Ganglioneuroblastoma*		*NF1* G722M
	32	6–10	*Gastrointestinal neuroectodermal tumor*		EWSR1-ATF1 fusion
	33	>16	*Alveolar rhabdomyosarcoma*		PAX3-FOXO1 fusion; amplification *MYCN*
	40	11–15	*Enchondroma*		
	43	11–15	*Osteochondroma*		
	47	11–15	*Ewing sarcoma*		
	48	0–5	*Adrenocortical carcinoma*		*CTNNB1* S37C; amplification *MYC, FAP*
	52	0–5	*Ependymoma*	III	
Hema	20	6–10	*Precursor T-lymphoblastic lymphoma*		Mismatch repair deficiency
	42	11–15	*Acute lymphoblastic leukemia*		*JAK2* R683S
	45	0–5	*Acute myeloid leukemia*		*WHSC1* E1099K

*Findings known prior to the comprehensive profiling are marked green*.

### Molecular profiling

DNA and RNA were extracted from the tumor samples stored in RNAlater using Qiagen's AllPrep DNA/RNA purification kit and QIAcube workstation. DNA from whole blood samples was isolated using Tecan's liquid handling automated station. Detailed method descriptions can be found in the Supplementary Material. Briefly, WES was performed on genomic and tumor DNA using Roche's KAPA HTP Library Preparation Kit and Agilent's SureSelectXT Clinical Research Exome kit. Paired-end sequencing was performed on Illumina instruments, with an average coverage of 50–100×. Data were processed using Qiagen's Biomedical Genomics Workbench and Ingenuity Variant Analysis. RNA sequencing was performed using Illumina's TruSeq Stranded Total RNA Library Prep Kit and paired-end sequencing was performed to gain an average output of 50–100 M reads. FusionMap was used for the screening of fusion transcripts [11], and if positive, validation was carried out by Sanger sequencing. Sequencing data can be found in ENA under accession number PRJEB23819. Somatic CNAs were detected by SNP arrays (CytoScan or OncoScan; Affymetrix, GSE108089). Data were analyzed using NEXUS (BioDiscovery). Expression levels were analyzed using Affymetrix's GeneChip® Human Genome U133 Plus 2.0 Array, with subsequent processing by Qlucore (Figure 1B).

### Clinical translation

A comprehensive diagnostic report integrating data obtained by the aforementioned methods and with suggestions for possible targeted interventions was available within 3–4 weeks from receiving the relevant biopsies and blood samples. The results were discussed at weekly multidisciplinary conferences attended by the responsible pediatric oncologist, an oncologist from the pediatric Phase I-II unit, pediatric surgeons, and representatives from relevant diagnostics units, i.e., Genomic Medicine, Pathology, and Diagnostic Imaging. The final clinical decision regarding a patient's inclusion into and/or recommendation for a particular targeted treatment protocol was made by the responsible pediatric oncologist, with agreement from the patient's parents.

For assessment of the efficacy of implemented advanced diagnostics, actionable molecular findings were reported. Findings were considered actionable if they (i) were relevant for diagnostics and prognostics (e.g., a known cancer driver mutation or amplification important for a patient's stratification, etc.), (ii) defined treatment targets for known anti-cancer drugs (not necessarily restricted to pediatric patients), or (iii) determined high-potential targets. Any finding belonging to one or more of these categories was assigned as actionable. The findings are summarized in detail in Table S2.

### Clinical trials available for pediatric cancers

Ongoing clinical studies for targeted therapy were retrieved from *ClinicalTrials.gov*. The search was performed using the search term “*cancer*” and eligibility criterion “*Child (birth*−*17)*.” A total of 8972 studies were found, of which 2037 were open on December 16, 2016. These studies were further filtered by “*Intervention*,” and 863 in which “*drug*” was a part of the study, were considered. Manual filtering of these 863 studies excluded ones (i) including adult cancers, (ii) related to transplantations, (iii) focusing on adjustments of standard treatment regimens, including radiation, (iv) aimed at renewed stratification of patients for standard treatments, and (v) on unspecified hematological malignancies; 152 studies remained for further assessment. A curated database of ongoing clinical trials in which targeted treatment options for pediatric cancer patients are available was subsequently generated (Table S3). Due to the source and filtration criteria there is a possibility that relevant clinical trials are missing.

## Results

### Patients and tumor parameters

The mean age at inclusion was 9.5 years (0–17). The male:female ratio was 1.4:1. Analyses were performed on 46 tumor samples (44 fresh-frozen and two formalin-fixed paraffin embedded), two bone marrow aspirates, three cerebrospinal fluid samples, and one archived DNA. Twenty patients had extracranial solid tumors and 25 had CNS tumors (Table 1). Three patients were diagnosed with a hematological malignancy, i.e., acute lymphoblastic leukemia, acute myeloid leukemia, or precursor T-lymphoblastic lymphoma. Eleven of the 25 CNS tumors underwent DNA methylation profiling (DKFZ Heidelberg) to obtain a second opinion on the diagnosis (for cohort details see Table 1 and Table S1). At the time of this review, six patients had passed away. None of the deceased patients had been included in neither clinical trials nor offered treatment based on actionable findings although they may have been included in other clinical studies.

### Overall achievements

The implementation of an optimized laboratory workflow comprising several analytical platforms resulted in final integrated diagnostic reports being available within 16.5 days (9–45 days). This ensured sufficient time for the multidisciplinary team and the pediatric Phase I–II unit to reach a decision regarding a patient's inclusion into a clinical trial. Moreover, when the DNA/RNA yield was limited, we took advantage of specialized analytic protocols, i.e., OncoScan, NEBNext, which led to a 100% (52/52) success rate for our patients' diagnostics (Figure 1B; Table S2).

A full molecular profiling on all the platforms, i.e., WES, RNA sequencing, expression arrays, and SNP arrays, was sought for each patient sample. However, full profiling was ultimately achieved for 40 of the 52 samples (77%). Profiling of the remaining patients was limited by the quality or quantity of the input material, or by the origin of the sample (formalin-fixed paraffin embedded, cerebrospinal fluid). All but one of the samples were analyzed by WES (*n* = 51; 98%). Forty-six samples were analyzed for CNAs by SNP array (88%), 42 by RNA sequencing (81%), and 43 by expression array (83%) (Figure 1C; Table 1).

**Figure 2:**
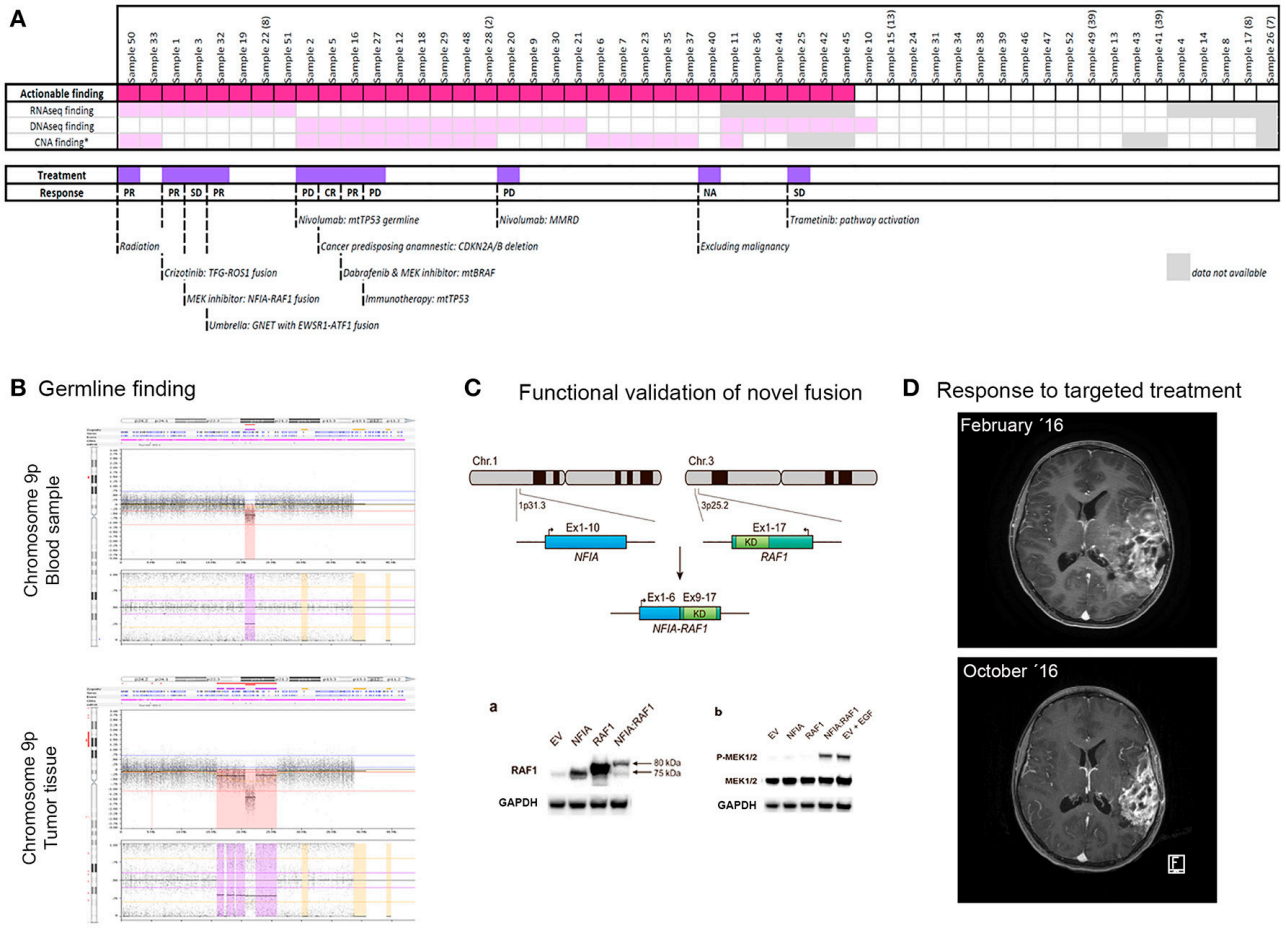
Overview of analyzed samples **(A)**, example of germline finding **(B)**, functional validation of novel fusion **(C)**, and extraordinary response to targeted treatment **(D)**. **(A)** Plot showing the distribution of findings per sample by individual methods, with the marking of samples with their treatment based on the findings. The response to treatment is also indicated. PR, partial response; CR, complete response; PD, progressive disease; SD, stable disease. **(B)** Germline hemizygous deletion in chromosome 9p21.3 involving *CDKN2A*. This finding was detected by SNP array in a 15-year-old girl with a history of several previous cancers of different origin. **(C)** Functional validation of novel gene fusion *NFIA-RAF1* detected in a 5-year-old boy with pilocytic/pilomyxoid astrocytoma showing activation of the MAPK pathway. Permission to reproduce the figure kindly granted by *Cancer Genetics* [12]. **(D)** MRIs of a 14-year-old girl with a *BRAF*-mutated tumor. The upper image shows the tumor at diagnosis and the lower image shows the tumor 8 months later after responding to targeted treatment (combination of dabrafenib and trametinib).

Actionable findings were definitively detected in 33 patients (63%) (Figure 2A; Table S2). Eighteen of the actionable findings proved relevant for an accurate diagnosis and 22 led to the identification of potential treatment targets. Eleven samples had a “double hit,” i.e., the finding had a diagnostic as well as a treatment impact. In nine out of the 33 patients, all (*n* = 3) or some of (*n* = 6) the findings were previously identified by histopathology. In four of these patients, gene re-arrangements were previously detected without knowing the fusion partner.

The most efficient method for identifying actionable findings was WES, detecting relevant mutations, including one germline mutation, in 39% of samples (20/51). Treatment- or diagnostic-relevant CNAs were detected in 37% of samples (17/46). In 10 samples (22%), detection of CNAs was not possible due to the low tumor burden in the sample. Strikingly, we identified a large number of fusions (9/42; 21%), including novel fusions relevant for treatment (e.g., *NFIA-RAF1* [12] and *TFG-ROS1* [13] in CNS tumors) and fusions specifying disease origin (e.g., *MN1-BEND2* for anaplastic ependymoma and *PAX3-FOXO1* for rhabdomyosarcoma).

### Clinical action based on molecular profiling

The final report presented to the pediatric oncologist always included all actionable findings, independent of their direct consequence for patient treatment. Hereby, all potential targets and drivers were disclosed and became theoretically reusable at later time points. Clinical intervention was implemented in 11 out of 44 patients (25%), i.e., two patients were diagnosed with a cancer-predisposing alteration, two patients were assigned the correct diagnosis, and eight patients received therapy based on the molecular findings (one of these patients was also included in the correct diagnosis group; Figure 2A). The most common therapy was immunotherapy, given to four patients. Two patients received MEK inhibitors, one patient received an ALK-ROS inhibitor, and one patient received combinatory treatment of BRAF- and MEK-inhibitors. Of note, all four patients treated with immune therapy experienced progressive disease.

If patients could be included in any of the 152 open clinical trials (Table S3), more than a half of the offered drugs would be the kinase inhibitors (*n* = 52) and antibodies (*n* = 33; Figure S1) in Phase I (*n* = 96) and Phase II (*n* = 64). Noticeably, 49 of the 152 open clinical trials involve target status for inclusion (Table S3). On this basis, we could include three patients with mutations in *ALK* and *BRAF*, respectively, and several patients in kinase inhibitor trials due to the alterations in relevant pathways.

### Patient cases

A 15-year-old girl with pleomorphic xanthoastrocytoma and a history of several previous cancers of different origin was examined for a potential cancer-predisposing alteration. SNP array disclosed a large hemizygous deletion in 9p21.3 involving *CDKN2A* (Figure 2B).

Two patients were included at primary diagnostics due to inconclusive results of standard diagnostics and thereby difficulties in assigning the correct treatment regimen. One of the patients, a 14-year-old girl (sample 16), was submitted for profiling based on suspicion of a rapidly progressing intracerebral angiosarcoma inferred from MRI data. Histologically, the sample resembled anaplastic pleomorphic xanthoastrocytoma/glioblastoma. DNA methylation analysis grouped the sample with pleomorphic xanthoastrocytoma/advanced stage ganglioglioma. Extended molecular profiling revealed somatic mutations in *BRAF* (p.V600E) and *ATRX* (p.R1739^*^), respectively. Based on these findings, the patient was treated with dabrafenib and trametinib and showed a partial response for more than 13 months (Figure 2D).

As mentioned above, a high proportion of patients with available RNA sequencing data were diagnosed with fusions (9/42). Notably, we identified two novel fusions, *NFIA-RAF1* and *TFG-ROS1*, both of which were used for the selection of targeted therapy. A 5-year-old boy was profiled due to a progressing brain tumor. Histologically, the tumor resembled pilocytic/pilomyxoid astrocytoma, WHO grade I-II. RNA sequencing identified an *NFIA-RAF1* fusion [12]. Subsequent functional analyses revealed a constitutive activation of the MAPK pathway (Figure 2C). The patient was therefore eligible for compassionate use treatment with the MEK inhibitor trametinib since he was not eligible for any open European MEK inhibitor trials. Radiologically, the tumor remained stable for almost 1 year; however, the disease was recently found to have clinically progressed. Treatment was discontinued due to the side-effects and severe neurological affection, and palliative care was initiated. The other novel fusion, *TFG-ROS1*, was found in a 6-year-old boy with a difficult-to-diagnose brain tumor which, for the most part, resembled atypical meningioma. The patient was eligible for crizotinib treatment and showed a partial response for more than 14 months [13].

## Discussion

In recent years, several studies have sought to map potential actionable aberrations and thereby optimize patient treatment [5–7, 9, 10, 14, 15]. Similar to these studies, high-throughput screening of pediatric tumors in our setting has proven to be very efficient, with 25% of patients experiencing direct benefits of the comprehensive genetic diagnostics. This took the form of targeted treatment or correct diagnosis, leading to an optimized treatment regimen for the patient. On the other hand, even though more than 60% of our samples were classified with an actionable finding, robust constraints encountered on the site of available and approved treatments greatly limit the utility of advanced diagnostics. However, a potential solution for rapidly deteriorating cases is the use of drugs for compassionate use, which has also proven to be efficient in our cohort.

At the time of this review, there were four Phase I/II studies conducting advanced molecular profiling in order to disclose molecular aberrations treatable with available drugs. For example, MOSCATO-01 study [16] and MATCH trial [17] (closed 2017) or ongoing CancerSCAN (NCT02638428). Most of the drugs target survival pathways in oncogenesis, for example via kinase inhibition (Figure S1), as despite having different aberrations, lesions often converge on common pathways. Therefore, pathway targeting might become a treatment option at earlier stages. However, in order to ensure the reliable identification of aberrations in targetable pathways, complex screening by several high-throughput methods would be required. However, not all centers currently possess the required infrastructure to pursue the comprehensive profiling. On the other hand, costs for analyses are continually decreasing, number of educated personal is increasing, and weighing those against the distress of side-effects and life-lasting sequelae caused by inefficient treatments, there is a lot of reasons to implement precision diagnostics in pediatric oncology.

A possible compromise on the costs and required complexity of several high-throughput analyses could be achieved by further development of the individual methods. For example, in the case of DNA sequencing, WES is becoming the first-choice method due to the fact that approximately 85% of disease-causing mutations reside in coding regions [18]. Furthermore, WES reads can be used to derive copy number changes, e.g. amplifications and deletions [19], and could consequently replace SNP array technologies. SNP arrays are however more robust for the screening of copy number changes under diagnostic settings, and can be designed to encompass specific mutations, e.g., OncoScan [20]. Nevertheless, DNA screening methods cannot currently detect gene fusions, which are of great significance in pediatric cancers [7, 9, 10, 15]. For that reason, RNA sequencing is essential for gene fusion identification, including novel fusions as shown in our cohort. Moreover, fusions have been shown to be very relevant indicators in the assignment of therapies. RNA sequencing can also be used to identify changes in the levels of transcripts and potentially for the detection of mutations, thus giving it the potential to become the first-choice method for pediatric cancer diagnostics. Apart from DNA and RNA analyses, there is also the whole field of epigenetics. DNA methylation changes have been shown to be crucial for CNS tumor classification [21], and there are undoubtedly many more epigenetic aberrations still to be discovered. In particular, epigenetic changes guiding the differentiation processes (e.g., polycomb complexes, histone deacetylases) are of great interest to pediatric oncologists [22–24].

In summary, the data collated during the initial period of the comprehensive molecular diagnostics clearly demonstrate the importance of high-throughput screening in pediatric oncology. Precision diagnostics have the potential to improve outcome and decrease side-effects of formerly curable pediatric cancers, either in conjunction with chemotherapy or by replacing some conventional agents. Moreover, as shown in this report, advanced diagnostics lead to better outcomes for children with high-risk tumors where progress has either been slow or has stalled. The potentialities suggested by this and other reports create new challenges for the field of pediatric oncology.

## Author contributions

OØ: collected the data, performed the analysis, and wrote the manuscript; AYS, MR, and FN: performed the genomic analysis; KN, RM, LH, TO, JS-R, BH, PW, HS, AMS or CR: were responsible for clinical part of the study and patients inclusion; KN: included patients into clinical trials; DS: performed the standard Diagnostics. All authors read and commented the manuscript and approved the data.

### Conflict of interest statement

The authors declare that the research was conducted in the absence of any commercial or financial relationships that could be construed as a potential conflict of interest.

## Supplementary material

Figure S1 Overview of 152 open clinical studies for pediatric cancers.

Table S1 Overview of patient and tumor parameters.

Table S2 Summary of all findings from WES, RNA sequencing, and SNP arrays.

Table S3 Summary of 152 open clinical trials for pediatric oncology (see Patients and Methods for details of filtering/criteria).
