# Supplementary figures and images for: Importance of Comprehensive Molecular Profiling for Clinical Outcome in Children With Recurrent Cancer
Source: Front Pediatr. 2018 Apr 20;6:114. doi: 10.3389/fped.2018.00114 (PMC5920151; doi:10.3389/fped.2018.00114)

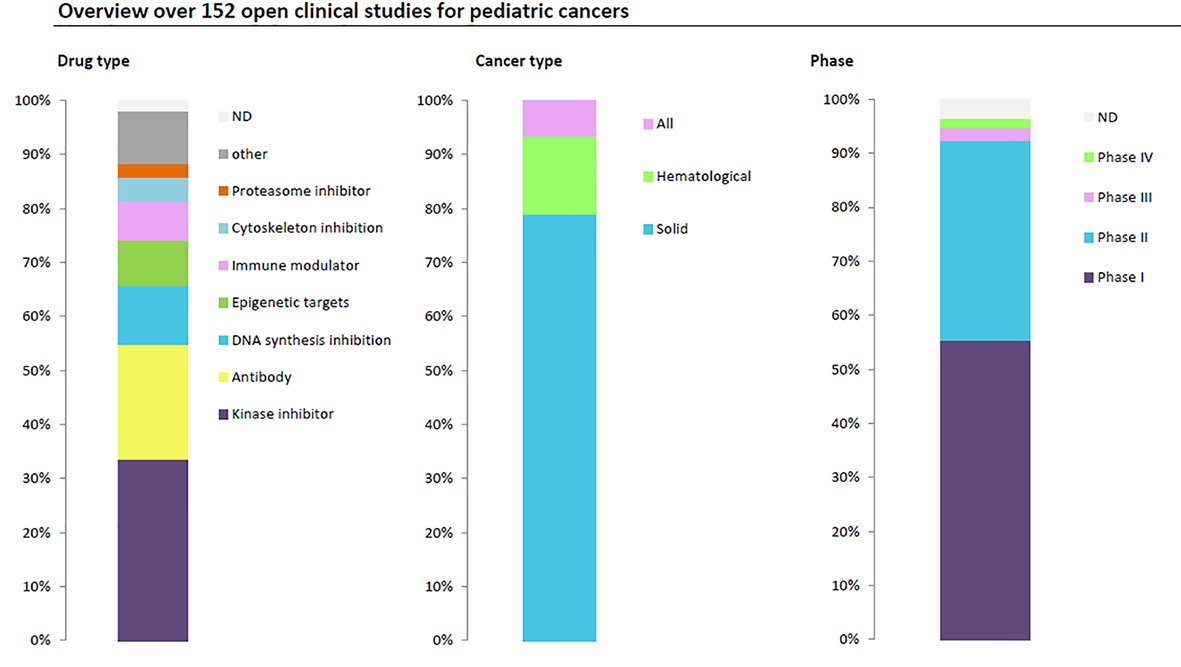

Supplement: Figure S1 — Overview of 152 open clinical studies for pediatric cancers. [file Image1.TIF]
