# Supplementary material for: Importance of Comprehensive Molecular Profiling for Clinical Outcome in Children With Recurrent Cancer
Source: Front Pediatr. 2018 Apr 20;6:114. doi: 10.3389/fped.2018.00114 (PMC5920151; doi:10.3389/fped.2018.00114)
Supplement: Table S1 — Overview of patient and tumor parameters. [file Table1.PDF]

Sample #	Patient re-inclusion	Gender	Age at inclusion (y)	Origin	Histopathological diagosis at inclusion	WHO grade	Classification based on DNAmet
Sample 1		male	6-10	tumor	Atypical meningioma	II	SEGA, WHO grad I
Sample 2		female	11-15	tumor	Choroid plexus carcinoma / Malignant peripheral nerve sheath tumor	III	Pleus choroideus carcinom
Sample 3		male	0-5	tumor	Pilocytic/pilomyxoid astrocytoma	I-II	Lavgrads midtliniegliom (pilocytisk astrocytom)
Sample 4		male	0-5	tumor	Glioblastoma	IV	
Sample 5		female	11-15	tumor	Pleomorphic xanthoastrocytoma	II-III	Pleomorft anthoastrocytom
Sample 6		male	0-5	tumor	Mesoblastic nephroma		
Sample 7		male	11-15	tumor	Signet ring cell carcinoma		
Sample 8		male	0-5	FFPE tumor	Ganglioglioma / Diffuse astrocytoma	I-II	Gangliogliom
Sample 9		female	>16	tumor	Chondrosarcoma		
Sample 10		male	11-15	tumor	Chordoma, dedifferentiated/anaplastic type (INI1-loss)		
Sample 11		female	11-15	FFPE tumor	Hepatoblastoma		
Sample 12		male	11-15	tumor	Diffuse midline glioma H3K27M-mutated	IV	
Sample 13		female	0-5	tumor	Anaplastic ependymoma	III	Cerebralt ependymom med RELA-fusion
Sample 14		female	6-10	tumor	Juvenile xanthogranuloma		
Sample 15	13	female	0-5	tumor	Anaplastic ependymoma	III	
Sample 16		female	11-15	tumor	Anaplastic pleomorfic xanthoastrocytoma / Glioblastoma	IV	Pleomorphic anthoastrocytoma /advanced stage ganglioglioma
Sample 17	8	male	0-5	tumor	Ganglioglioma / Diffuse astrocytoma	I-II	
Sample 18		female	6-10	tumor	Diffuse midline glioma H3K27M-mutated		
Sample 19		male	11-15	tumor	Alveolar rhabdomyosarcoma		
Sample 20		male	6-10	tumor	Precursor T-lymphoblastic lymphoma		
Sample 21	20	male	6-10	tumor	Glioblastoma	IV	
Sample 22	8	male	0-5	tumor	Ganglioglioma / Diffuse astrocytoma	I-II	
Sample 23		female	>16	tumor	Malignant peripheral nerve sheath tumor		
Sample 24		male	6-10	tumor	Pilocytic astrocytoma	I	Pilocytic astrocytoma WHO grade I
Sample 25		female	>16	DNA	Pilocytic/pilomyxoid astrocytoma	I-II	
Sample 26	7	male	11-15	tumor	Signet ring cell carcinoma; immune therapy screening		
Sample 27		female	>16	tumor	Nephroblastoma		
Sample 28	2	female	11-15	tumor	Choroid plexus carcinoma / Malignant peripheral nerve sheath tumor	III	
Sample 29		male	0-5	tumor	Neuroblastoma		
Sample 30		female	0-5	tumor	Ganglioneuroblastoma		
Sample 31		male	0-5	tumor	Anaplastic ependymoma	III	Undefined, highest score for grp A ependymoma of posterior fossa
Sample 32		male	6-10	tumor	Gastrointestinal neuroectodermal tumor		
Sample 33		male	>16	tumor	Alveolar rhabdomyosarcoma		
Sample 34		female	>16	tumor	Atypical neurocytoma	II	
Sample 35		male	11-15	tumor	Chondroblastic osteosarcoma		
Sample 36		female	0-5	tumor	Astrocytoma	NA	Low-grade glioma, pilocytic astrocytoma
Sample 37		male	0-5	tumor	Neuroblastoma		
Sample 38		male	0-5	tumor	Anaplastic ependymoma	III	Group A ependymoma of the posterior fossa
Sample 39		male	11-15	CSF_DNA	Pineoblastoma	IV	
Sample 40		male	11-15	tumor	Enchodromas		
Sample 41	39	male	11-15	CSF_RNA	Pineoblastoma	IV	
Sample 42		male	11-15	marrow	ALL		
Sample 43		female	11-15	tumor	Osteochondroma		
Sample 44		male	11-15	tumor	Diffuse astrocytoma	II	
Sample 45		male	0-5	marrow	AML recidiv		
Sample 46		male	11-15	tumor	Malignant peripheral nerve sheeth tumor		
Sample 47		male	11-15	tumor	Ewing sarcoma		
Sample 48		female	0-5	tumor	Adrenocortical carcinoma		
Sample 49	39	male	11-15	CSF	Pineoblastoma	IV	
Sample 50		female	6-10	tumor	Anaplastic ependymoma	III	
Sample 51		female	>16	tumor	Rhabdomyosarcoma		
Sample 52		male	0-5	tumor	Ependymoma	III	
