# Supplementary material for: Importance of Comprehensive Molecular Profiling for Clinical Outcome in Children With Recurrent Cancer
Source: Front Pediatr. 2018 Apr 20;6:114. doi: 10.3389/fped.2018.00114 (PMC5920151; doi:10.3389/fped.2018.00114)
Supplement: Table S2 — Summary of all findings from WES, RNA sequencing, and SNP arrays. [file Table2.PDF]

| Sample # | Patient re-inclusion | Whole exome sequencing | | SNP arrays_Copy number alterations | | | | | RNAsequencing |
|---|---|---|---|---|---|---|---|---|---|
| | | **WES_somatic mutations** | **WES_germline** | **LOH** | **Homozygous deletion** | **Amplification** | **D+LOH** | **Other** | **Fusion** |
| Sample 1 | | 0 | x | x | x | x | x | | TFG-ROS1 |
| Sample 2 | | 0 | *TP53* c.742C>T p.R248W | HRD | RB1 | MET, CDK6, NOTCH2 | x | | NF |
| Sample 3 | | 0 | x | x | x | x | x | | NFIA-RAF1 |
| Sample 4 | | 0 | x | germline | x | x | x | | no available |
| Sample 5 | | BRAF V600 | 0 | germline | CDKN2A/B | | CDKN2A/B germline | | NF |
| Sample 6 | | 0 | x | x | x | ERVV1, ERVV2 | x | | NF |
| Sample 7 | | 0 | x | x | x | x | x | germline gain 6q27 | NF |
| Sample 8 | | 0 | x | x | x | x | x | low tumor burden | no available |
| Sample 9 | | IDH1 c.395G>T p.Ag132Leu | x | x | x | x | x | low tumor burden | NF |
| Sample 10 | | PIK3CG c3172GZC p.G1058R | x | x | x | x | x | subclonality | NF |
| Sample 11 | | CDKN2A c223_234delTC, p.L78fs*41 | x | x | x | x | CDKN2A/B | | no available |
| Sample 12 | | H3F3A/B c.83A>T, p.Lys28Met; TP53 c.469_471delGT | x | x | x | ch7, ch22 | x | | NF |
| Sample 13 | | 0 | x | x | x | x | x | unstable ch11 | NF |
| Sample 14 | | 0 | x | x | x | x | x | low tumor burden | no available |
| Sample 15 | 13 | 0 | x | x | x | x | x | unstable ch11, gain ch19 and X | NF |
| Sample 16 | | BRAF c.1799T>A, p.V600E; ATRX c.5215C>T, p.R1739* | 0 | ch6,8,9,10,18,21 | CDKN2A/B | x | x | germline withour positive findings | NF |
| Sample 17 | 8 | normal væv | x | x | x | x | x | low tumor burden | FGFR3-TACC3 |
| Sample 18 | | H3F3A/B c.83A>T, p.K28M; TP53 c.401T>C, p.F134S a | x | x | x | x | ch16, ch22 | unstable ch11, 12 and 21 | NF |
| Sample 19 | | 0 | x | x | x | x | x | low tumor burden | PAX3-FOXO1 |
| Sample 20 | | MMRD_FBXW7 x2 mutationer | x | x | x | x | x | unstable ch9 and ch14 | NF |
| Sample 21 | 20 | PTEN (c.800dupA, p.D268fs*30); ABCC2 (c.1177C>T, p | x | x | x | x | x | | NF |
| Sample 22 | 8 | 0 | x | x | x | x | x | low tumor burden | FGFR3-TACC3 |
| Sample 23 | | 0 | x | x | x | FGFR1 | CDKN2A/B | unstable, subclonality | NF |
| Sample 24 | | 0 | x | x | x | x | x | | NF |
| Sample 25 | | FGFR1 p.N546K, PTPN11 p.E69K | x | o | o | o | o | | no available |
| Sample 26 | 7 | x | x | o | o | o | o | | no available |
| Sample 27 | | TP53 c.75-1G>C, splice site loss; *GNA11* p.R183C | x | x | x | NGFR | HOXB family, ch7 | | NF |
| Sample 28 | 2 | 0 | *TP53* c.742C>T p.R248W | HRD | RB1 | MET, CDK6, NOTCH2 | x | | NF |
| Sample 29 | | ALK c.3522C>A, p.F1174L | 0 | 1p | x | MYCN | x | NB specific CNAs | NF |
| Sample 30 | | NF1 p.G722M | 0 | x | x | x | x | unstable | NF |
| Sample 31 | | 0 | x | x | x | x | x | low tumor burden | NF |
| Sample 32 | | 0 | x | x | x | x | x | unstable ch6 | EWSR1-ATF1 |
| Sample 33 | | 0 | x | x | x | MYCN, 1q21.3 | CDKN2A/B | | PAX3-FOXO1 |
| Sample 34 | | 0 | x | x | x | x | x | low tumor burden | NF |
| Sample 35 | | 0 | x | HRD | x | multiple | multiple | unstable | NF |
| Sample 36 | | KRAS p.E63K | x | x | x | x | x | trizomi ch2, 9, 11, subclonality | no available |
| Sample 37 | | 0 | x | UPD | x | MET, JAG1 | CDKN2A/B, PTEN | unstable | NF |
| Sample 38 | | 0 | x | x | x | x | x | unstable | NF |
| Sample 39 | | 0 | x | x | x | x | x | unstable | NF |
| Sample 40 | | 0 | x | x | x | x | x | low tumor burden | NF |
| Sample 41 | 39 | 0 | x | o | o | o | o | not analyzed | NF |
| Sample 42 | | JAK2 c.20494A>T, p.Arg683Ser | x | o | o | o | o | not analyzed | no available |
| Sample 43 | | 0 | x | o | o | o | o | not analyzed | NF |
| Sample 44 | | OBS_MMRD; PIK3CA p.V344M; VHL p.E160K;BRACA1 | x | x | x | x | x | trizomi ch6, 7, 9, 11, 12, 20 | no available |
| Sample 45 | | WHSC1 p.E1099K | | o | o | o | o | not analyzed | no available |
| Sample 46 | | 0 | x | x | x | x | x | low tumor burden | NF |
| Sample 47 | | 0 | x | x | x | x | x | unstable, subclonality | NF |
| Sample 48 | | CTNNB1 p.Ser37Cys | x | x | x | MYC, FAP | APC | unstable | NF |
| Sample 49 | 39 | 0 | x | x | x | x | x | suboptimal quality | NF |
| Sample 50 | | 0 | x | x | chX | x | NF2, ESWR1 | monozomi ch2, 4, 8, 10, 14, 16, 18 | MN1-BEND2 |
| Sample 51 | | 0 | x | x | x | x | x | unstable | PAX3-FOXO1 |
| Sample 52 | | 0 | x | x | x | x | x | monozomi ch3, 6, 10 | NF |
