# Supplementary material for: Importance of Comprehensive Molecular Profiling for Clinical Outcome in Children With Recurrent Cancer
Source: Front Pediatr. 2018 Apr 20;6:114. doi: 10.3389/fped.2018.00114 (PMC5920151; doi:10.3389/fped.2018.00114)
Supplement: Table S3 — Summary of 152 open clinical trials for pediatric oncology (see Patients and Methods for details of filtering/criteria). [file Table3.PDF]

URL	Targets	Drug	Drug type/class	Cancer (solid/hemo)	Phase	Inclusion based on target status
https://ClinicalTrials.gov/show/NCT02465528	ALK	*Ceritinib (LDK378)*	kinase inhibitor	solid	2	ALK positive (ALK+)
https://ClinicalTrials.gov/show/NCT01606878	ALK and ROS1 inhibitor	*Crizotinib*	kinase inhibitor	solid	1	no
https://ClinicalTrials.gov/show/NCT01742286	ALK inhibitor	*LDK378*	kinase inhibitor	solid	1	ALK aberrations
https://ClinicalTrials.gov/show/NCT02729961	ALK inhibitor, anti-CD30-antibody	*Ceritinib, Brentuximab Vedotin*	kinase inhibitor	solid	1,2	ALK and CD30 positive
https://ClinicalTrials.gov/show/NCT02780128	ALK inhibitor, RAS-MAPK inhibitor,	*Ceritinib, Trametinib, HDM201*	kinase inhibitor	solid	1	advanced molecular profiling
https://ClinicalTrials.gov/show/NCT01644773	ALK/ROS and c-kit inhibitor	*Crizotinib and Dasatinib*	kinase inhibitor	solid	1	no
https://ClinicalTrials.gov/show/NCT02982941	anti-B7-H3 antibody	*Enoblituzumab*	antibody	solid	1	B7-H3 positive by IHC
https://ClinicalTrials.gov/show/NCT02979899	anti-CD105-antibody, VEGFR inhib	*TRC105, PAZOPANIB*	antibody	solid	3	no
https://ClinicalTrials.gov/show/NCT02187354	anti-CD19-antigen	*Blinatumomab*	antibody	hemo	4	no
https://ClinicalTrials.gov/show/NCT02393859	anti-CD19-antigen	*Blinatumomab*	antibody	hemo	3	Philadelphia negative
https://ClinicalTrials.gov/show/NCT01516580	anti-CD20-antibody	*Rituximab*	antibody	hemo	3	no
https://ClinicalTrials.gov/show/NCT02343406	anti-EGFR-antibody	*ABT-414*	antibody	solid	2	EGFR amp
https://ClinicalTrials.gov/show/NCT02107963	anti-GD2-antibody	*Anti-GD2-CAR engineered T cells*	antibody	solid	1	GD2+ expression by ICH
https://ClinicalTrials.gov/show/NCT00877110	anti-GD2-antibody	*Anti-GD2 3F8 Antibody*	antibody	solid	1	high risk NB
https://ClinicalTrials.gov/show/NCT01711554	anti-GD2-antibody	*Dinutuximab*	antibody	solid	1	high-risk NB
https://ClinicalTrials.gov/show/NCT02650648	anti-GD2-antibody	*Hu3F8*	antibody	solid	1	high-risk NB
https://ClinicalTrials.gov/show/NCT02100930	anti-GD2-antibody	*Hu3F8*	antibody	solid	1	high-risk NB
https://ClinicalTrials.gov/show/NCT02641782	anti-GD2-antibody	*antibody ch14.18*	antibody	solid	2	high-risk NB
https://ClinicalTrials.gov/show/NCT02693171	anti-GD2-antibody	*Dinutuximab*	antibody	solid	4	high-risk NB
https://ClinicalTrials.gov/show/NCT02169609	anti-GD2-antibody	*Dinutuximab. Immunotherapy*	antibody	solid	2	high-risk NB
https://ClinicalTrials.gov/show/NCT02743429	anti-GD2-antibody	*dinutuximab beta*	antibody	solid	2	no
https://ClinicalTrials.gov/show/NCT02914405	anti-PD1-antibody	*Nivolumab*	antibody	solid	1	high-risk NB
https://ClinicalTrials.gov/show/NCT02901145	anti-PD1-antibody	*Nivolumab*	antibody	solid	1,2	no
https://ClinicalTrials.gov/show/NCT02961101	anti-PD1-antibody	*Anti-PD-1 antibody*	antibody	solid	1,2	no
https://ClinicalTrials.gov/show/NCT02775292	anti-PD1-antibody	*Nivolumab*	antibody	solid	1	NY-ESO-1 positive (IHC), HLA-A2.1 positivity by molecular subtyping
https://ClinicalTrials.gov/show/NCT02793466	anti-PD-L1 antibody	*Durvalumab*	antibody	solid	1	no
https://ClinicalTrials.gov/show/NCT02541604	anti-PD-L1 antibody	*MPDL3280A*	antibody	solid	1,2	no
https://ClinicalTrials.gov/show/NCT02748135	anti-PIGF-antibody	*TB-403*	antibody	solid	1,2	no
https://ClinicalTrials.gov/show/NCT02114229	aurora A kinase inhibitor	*alisertib*	kinase inhibitor	solid	2	loss of SMARCB1/BAF47 by iCH or by mol methods
https://ClinicalTrials.gov/show/NCT02596828	BCR-ABL TK and mTOR inhibitor	*Dasatinib, Rapamycin*	kinase inhibitor	solid	2	no
https://ClinicalTrials.gov/show/NCT02897986	beta blockator, tubulin blockage	*propranolol, vinorelbine*	cytoskeleton	solid	1	no
https://ClinicalTrials.gov/show/NCT01677741	BRAF V600	*Dabrafenib*	antibody	solid	1	BRAF V600 mutation
https://ClinicalTrials.gov/show/NCT02124772	BRAF V600	*Trametinib, Dabrafenib*	antibody	solid	1	no
https://ClinicalTrials.gov/show/NCT01748149	BRAF V600E	*Vemurafenib*	antibody	solid	0	BRAFV600E or BRAF Ins T mutation
https://ClinicalTrials.gov/show/NCT02644460	CDK4/6 inhibitor	*Abemaciclib*	kinase inhibitor	solid	1	no
https://ClinicalTrials.gov/show/NCT02163356	ceramide accumulation	*Fenretinide Lym-X-Sorb*	other	solid	1	high-risk NB
https://ClinicalTrials.gov/show/NCT01187810	ceramide accumulation	*Fenretinide*	other	hemo	1	no
https://ClinicalTrials.gov/show/NCT02075177	ceramide accumulation	*Fenretinide Lym-X-Sorb*	other	solid	ND	no
https://ClinicalTrials.gov/show/NCT02808650	CHK1/2 Inhibitor	*LY2606368*	kinase inhibitor	solid	1	no
https://ClinicalTrials.gov/show/NCT02177825	c-kit inhibitor	*Imatinib Mesylate*	kinase inhibitor	solid	2	NF1
https://ClinicalTrials.gov/show/NCT02233049	c-kit, EGFR-TK, mTOR inhibitor	*Erlotinib, Everolimus, Dasatinib*	kinase inhibitor	solid	2	EGFR expression, PTEN loss
https://ClinicalTrials.gov/show/NCT01491763	c-kit, PDGFR, abl inhibitor	*Imatinib*	kinase inhibitor	hemo	4	BCR-ABL
https://ClinicalTrials.gov/show/NCT02885324	c-Met, VEGFR2 inhibitor	*Cabozantinib*	kinase inhibitor	solid	2	no
https://ClinicalTrials.gov/show/NCT02630043	COMT inhibitor	*Tolcapone*	other	solid	1	no
https://ClinicalTrials.gov/show/NCT02323880	CRM1 inhibitor (exportin1)	*Selinexor*	other	solid	1	no
https://ClinicalTrials.gov/show/NCT02390752	CSF1R inhibitor	*PLX3397*	kinase inhibitor	solid/hemo	1,2	no
https://ClinicalTrials.gov/show/NCT02499861	cytidine analog, DNAmethylation a	*Decitabine and Genistein*	DNA, epigenetic drug	solid/hemo	1,2	no
https://ClinicalTrials.gov/show/NCT02333058	DNA alkylation	*Treosulfan*	DNA	hemo	2	no
https://ClinicalTrials.gov/show/NCT02889445	DNA alkylation	*4-Demethyl-4-cholesteryloxycarbo*	DNA	solid	1	no
https://ClinicalTrials.gov/show/NCT01900509	DNA alkylation	*Bendamustine*	DNA	solid/hemo	1	no
https://ClinicalTrials.gov/show/NCT00601003	DNA breakage	*Nifurtimox*	DNA	solid	2	no
https://ClinicalTrials.gov/show/NCT02828358	DNA methylation	*Azacitidine*	epigenetic drug	hemo	ND	confirmed diagnosis; gene re-arrangments
https://ClinicalTrials.gov/show/NCT02447666	DNA methylation	*Azacitidine*	epigenetic drug	hemo	2	no
https://ClinicalTrials.gov/show/NCT02450877	DNA methylation	*Azacitidine*	epigenetic drug	hemo	2	t(8;21),RUNX1-RUNX1T1; inv(16), CBFb/MYH11; t(9;11), MLL-AF9; NPM1 mutation;FLT3-ITD mutation
https://ClinicalTrials.gov/show/NCT01191541	DNA synthesis	*Cytarabine*	DNA	hemo	ND	no
https://ClinicalTrials.gov/show/NCT02724163	DNA synthesis	*Liposomal daunorubicin*	DNA	hemo	3	no
https://ClinicalTrials.gov/show/NCT02013336	DNA synthesis	*MM-398 Plus Cyclophosphamide*	DNA	solid	1	no

https://ClinicalTrials.gov/show/NCT02425904	DNA synthesis	*Clofarabine*	DNA	solid	2	no
https://ClinicalTrials.gov/show/NCT00983398	DNA synthesis	*Melphalan*	DNA	solid	1, 2	no
https://ClinicalTrials.gov/show/NCT01943682	DNA synthesis	*CPX-351*	DNA	solid/hemo	1	no
https://ClinicalTrials.gov/show/NCT01884740	EGFR and VEGFA inhibitor	*Erbitux and Bevacizumab*	antibody	solid	1,2	no
https://ClinicalTrials.gov/show/NCT02687386	EGFR inhibitor	*Mitoxantrone packaged EDV*	other	solid	1	EGFR expression positive
https://ClinicalTrials.gov/show/NCT02672241	EGFR inhibitor	*Nimotuzumab*	antibody	solid	2	no
https://ClinicalTrials.gov/show/NCT02372006	EGFR/HER2 inhibitor	*afatinib*	kinase inhibitor	solid	1	ErbB deregulation
https://ClinicalTrials.gov/show/NCT02934256	EGFR-TK inhibitor	*Icotinib*	kinase inhibitor	solid	2	NF2
https://ClinicalTrials.gov/show/NCT02624388	EGFR-TK inhibitor	*Genistein*	kinase inhibitor	solid	2	no
https://ClinicalTrials.gov/show/NCT02689336	EGFR-TK inhibitor	*Erlotinib*	kinase inhibitor	solid	2	non-synonymous mutation in EGFR, ERBB2, or JAK2V617F (JAK2)
https://ClinicalTrials.gov/show/NCT02615106	Endostatin	*Endostar*	other	solid	2	no
https://ClinicalTrials.gov/show/NCT02657005	EWS-FLI1 inhibitor	*TK216*	other	solid	1	no
https://ClinicalTrials.gov/show/NCT02601937	EZH2 inhibitor	*Tazemetostat*	epigenetic drug	solid	1	no
https://ClinicalTrials.gov/show/NCT02676323	HDAC inhibitor	*Panobinostat*	epigenetic drug	hemo	1	no
https://ClinicalTrials.gov/show/NCT02780804	HDAC inhibitor	*Entinostat*	epigenetic drug	solid	1	no
https://ClinicalTrials.gov/show/NCT02899715	HDAC inhibitor	*Panobinostat*	epigenetic drug	solid	1	no
https://ClinicalTrials.gov/show/NCT02717455	HDAC inhibitor	*Panobinostat*	epigenetic drug	solid	1	no
https://ClinicalTrials.gov/show/NCT02420613	HDAC inhibitor	*SAHA*	epigenetic drug	solid	1	no
https://ClinicalTrials.gov/show/NCT02774421	HER2 inhibitor	*Trastuzumab*	antibody	solid	1	no
https://ClinicalTrials.gov/show/NCT02502708	IDO pathway inhibitor	*Indoximod*	other	soldi	1	no
https://ClinicalTrials.gov/show/NCT02538965	IKZF1/3 degradation	*Lenalidomide*	other	hemo	2	no
https://ClinicalTrials.gov/show/NCT02906371	IL-6R antibody, programmed T-cell	*Tocilizumab, CART 19*	antibody	hemo	ND	CD19 pos ALL
https://ClinicalTrials.gov/show/NCT02511132	immunomodulation	*Vigil*	immune system modulator	solid	2	EWS translocation
https://ClinicalTrials.gov/show/NCT01795313	immunomodulation	*Imiquimod*	immune system modulator	solid	ND	HLA-A2 positive based on flow cytometry
https://ClinicalTrials.gov/show/NCT02756845	immunomodulation	*Talimogene Laherparepvec*	immune system modulator	solid	1	HSV-1 serostatus
https://ClinicalTrials.gov/show/NCT01860937	immunomodulation	*leukapheresis or collection of PBM*	immune system modulator	hemo	1	no
https://ClinicalTrials.gov/show/NCT01803152	immunomodulation	*Dendritic Cell Vaccine*	immune system modulator	solid	1	no
https://ClinicalTrials.gov/show/NCT02750891	immunomodulation	*DSP-7888*	immune system modulator	solid	1,2	no
https://ClinicalTrials.gov/show/NCT02573896	immunomodulation	*Immunotherapy*	immune system modulator	solid	1	no
https://ClinicalTrials.gov/show/NCT01188096	immunomodulation	*Poly-ICLC*	immune system modulator	solid	2	no
https://ClinicalTrials.gov/show/NCT02761915	immunomodulation	*1RG-CART*	immune system modulator	solid	1	no
https://ClinicalTrials.gov/show/NCT02415153	immunomodulation	*Pomalidomide*	immune system modulator	solid	1	no
https://ClinicalTrials.gov/show/NCT01528046	insulin resistance	*Metformin*	other	solid	1	no
https://ClinicalTrials.gov/show/NCT02343224	JAK-STAT pathway	*Pegylated interferon alpha-2b*	immune system modulator	solid	2	no
https://ClinicalTrials.gov/show/NCT02639546	MEK inhibitor	*Cobimetinib*	kinase inhibitor	solid	1,2	known or expected RAS/RAF/MEK/ERK pathway involvement
https://ClinicalTrials.gov/show/NCT02285439	MEK inhibitor	*MEK162*	kinase inhibitor	solid/hemo	1,2	known or presumed Ras-Raf pathway activation
https://ClinicalTrials.gov/show/NCT01362803	MEK inhibitor	*AZD6244*	kinase inhibitor	solid	1,2	NF1
https://ClinicalTrials.gov/show/NCT01858155	melatonin	*Melatonin*	other	solid	1	no
https://ClinicalTrials.gov/show/NCT02197637	microtubules inhibitor	*VINORELBINE*	cytoskeleton	solid	2	no
https://ClinicalTrials.gov/show/NCT01837862	microtubules inhibitor	*Mebendazole*	cytoskeleton	solid	1,2	no
https://ClinicalTrials.gov/show/NCT02171260	microtubules inhibitor	*Eribulin Mesilate*	cytoskeleton	solid	1	no
https://ClinicalTrials.gov/show/NCT02596503	microtubules inhibitor	*Eribulin*	cytoskeleton	solid	1	no
https://ClinicalTrials.gov/show/NCT02644291	microtubules inhibitor	*Mebendazole*	cytoskeleton	solid	1	no
https://ClinicalTrials.gov/show/NCT01962103	microtubules inhibitor	*nab-paclitaxel*	cytoskeleton	solid	1,2	no
https://ClinicalTrials.gov/show/NCT01467986	mTOR and multi TK inhibitor	*Dasatinib, Rapamycin*	kinase inhibitor	solid	2	high-risk NB
https://ClinicalTrials.gov/show/NCT01582191	mTOR and multikinase inhibitor	*Vandetanib and Everolimus*	kinase inhibitor	solid	1	no
https://ClinicalTrials.gov/show/NCT01265030	mTOR inhibitor	*Sirolimus*	kinase inhibitor	solid	1,2	mTOR pathway activated desmoid tumor
https://ClinicalTrials.gov/show/NCT01345136	mTOR inhibitor	*RAD001*	kinase inhibitor	solid	2	NF2
https://ClinicalTrials.gov/show/NCT01614197	mTOR inhibitor	*Temsirolimus*	kinase inhibitor	hemo	1	no
https://ClinicalTrials.gov/show/NCT01523977	mTOR inhibitor	*Everolimus*	kinase inhibitor	hemo	1	no
https://ClinicalTrials.gov/show/NCT02574728	mTOR inhibitor	*Sirolimus*	kinase inhibitor	solid	2	no
https://ClinicalTrials.gov/show/NCT02155920	mTOR inhibitor	*Everolimus*	kinase inhibitor	solid	2	no
https://ClinicalTrials.gov/show/NCT02343718	mTOR inhibitor	*Vinblastine and Temsirolimus*	kinase inhibitor	solid	1	no
https://ClinicalTrials.gov/show/NCT01529593	mTOR inhibitors	*Temsirolimus and Metformin*	kinase inhibitor	solid	1	no
https://ClinicalTrials.gov/show/NCT02867592	multi TK inhibitor	*Cabozantinib S-malate*	kinase inhibitor	solid	2	no
https://ClinicalTrials.gov/show/NCT02932280	multikinase inhibitor (EGFR and HE	*Neratinib*	kinase inhibitor	solid/hemo	1,2	no
https://ClinicalTrials.gov/show/NCT01962896	multikinase inhibitor (EGFR and m	*Erlotinib, Sirolimus*	kinase inhibitor	solid	2	EGFR and mTOR pathway activation assessment
https://ClinicalTrials.gov/show/NCT01884922	multikinase inhibitor (PDGFR, c-KIT	*Nilotinib*	kinase inhibitor	solid	1	no
https://ClinicalTrials.gov/show/NCT01396148	multikinase inhibitor (PDGFR, VEGI	*sunitinib*	kinase inhibitor	solid	2	no

https://ClinicalTrials.gov/show/NCT02030964	ODC inhibitor	*DFMO*	epigenetic drug	solid	1	no
https://ClinicalTrials.gov/show/NCT02044120	PARP inhibitor	*Niraparib*	DNA	solid	1	no
https://ClinicalTrials.gov/show/NCT02392793	PARP inhibitor	*Talazoparib*	DNA	solid	1	no
https://ClinicalTrials.gov/show/NCT01858168	PARP inhibitor, DNA alkylation	*Olaparib and Temozolomide*	DNA	solid	1	no
https://ClinicalTrials.gov/show/NCT02116777	PARP inhibitor, DNA alkylation	*Talazoparib, Temozolomide*	DNA	solid/hemo	1,2	no
https://ClinicalTrials.gov/show/NCT02389309	PDGFR and SRC Inhibitor	*Dasatinib, Cyclophosphamide, Ten*	kinase inhibitor	solid	1	no
https://ClinicalTrials.gov/show/NCT02677116	PDGFRA antibody	*Olaratumab*	antibody	solid	1	no
https://ClinicalTrials.gov/show/NCT01887522	PDGFRA, c-kit inhibitor	*Vinblastine + Nilotinib*	kinase inhibitor	solid	2	no
https://ClinicalTrials.gov/show/NCT02909777	PI3K/HDAC inhibitor	*CUDC-907*	kinase inhibitor, epigenetic	solid	1	no
https://ClinicalTrials.gov/show/NCT02722135	PLK1 inhibitor	*Volasertib*	kinase inhibitor	hemo	1	no
https://ClinicalTrials.gov/show/NCT02419755	proteasome and HDAC inhibitor	*Bortezomib and Vorinostat*	proteasome inhibitor; epige	hemo	2	positive for MLL
https://ClinicalTrials.gov/show/NCT02535806	proteasome inhibitor	*Bortezomib*	proteasome inhibitor	hemo	2	no
https://ClinicalTrials.gov/show/NCT02139397	proteasome inhibitor	*Bortezomib*	proteasome inhibitor	solid	1,2	no
https://ClinicalTrials.gov/show/NCT02512926	proteasome inhibitor	*Carfilzomib*	proteasome inhibitor	solid/hemo	1	no
https://ClinicalTrials.gov/show/NCT02015728	specific	*Etoposide, Sorafenib,Everolimus,Er*	ND	solid	ND	advanced molecular profiling
https://ClinicalTrials.gov/show/NCT02638428	specific	*17 drugs*	ND	solid/hemo	2	CancerSCAN NGS positiv detection
https://ClinicalTrials.gov/show/NCT02390843	STAT3 inhibitor	*Simvastatin*	other	solid	1	no
https://ClinicalTrials.gov/show/NCT02813135	target specific	*MOSCATO-01*	ND	solid/hemo	1,2	advanced molecular profiling
https://ClinicalTrials.gov/show/NCT02800889	topoisomerase II inhibitor	*Pixantrone*	DNA	solid	1	no
https://ClinicalTrials.gov/show/NCT02303028	topoizomerase and multikinase inh	*Topotecan and Pazopanib*	DNA	solid	1,2	no
https://ClinicalTrials.gov/show/NCT02354547	TP53 mimic	*SGT-53*	other	solid	1	no
https://ClinicalTrials.gov/show/NCT02637687	TRK Inhibitor	*LOXO-101*	kinase inhibitor	solid	1	no
https://ClinicalTrials.gov/show/NCT02650401	Trk, ROS1, ALK inhibitor	*Entrectinib*	kinase inhibitor	solid	1	NTRK1/2/3, ROS1, or ALK gene rearrangements
https://ClinicalTrials.gov/show/NCT02164838	VEGF inhibitor	*Axitinib*	kinase inhibitor	solid	1	no
https://ClinicalTrials.gov/show/NCT01956669	VEGF inhibitor	*Pazopanib GW786034*	kinase inhibitor	solid	2	no
https://ClinicalTrials.gov/show/NCT02446431	VEGF inhibitor, DNA alkylation, HD	*Metronomic Therapy (Becacizumal*	kinase inhibitor	solid	0	no
https://ClinicalTrials.gov/show/NCT01767792	VEGFA inhibitor	*Bevacizumab*	antibody	solid	2	NF2
https://ClinicalTrials.gov/show/NCT01356290	VEGFA inhibitor	*bevacizumab*	antibody	solid	2	no
https://ClinicalTrials.gov/show/NCT02298348	VEGFR, PDGFR and Raf kinase inhil	*Sorafenib*	kinase inhibitor	solid	1	high-risk NB
https://ClinicalTrials.gov/show/NCT02747537	VEGFR, PDGFR and Raf kinase inhil	*Sorafenib*	kinase inhibitor	solid	2	non-synonymous mutation in Raf, PDGFR, VEGFR, Flt-3, KIT, JAK, STAT, RAS, MEK, or ERK
https://ClinicalTrials.gov/show/NCT02432274	VEGFR1/2/3 inhibitor	*Lenvatinib*	kinase inhibitor	solid	1,2	no
https://ClinicalTrials.gov/show/NCT02564198	VEGFR2 inhibitor	*Ramucirumab*	antibody	solid	1	no
https://ClinicalTrials.gov/show/NCT01391962	VEGFR-TK inhibitor	*Sunitinib, Cediranib*	kinase inhibitor	solid	2	no
https://ClinicalTrials.gov/show/NCT02791919	Wee1 Kinase Inhibitor	*AZD1775*	kinase inhibitor	hemo	1	no
https://ClinicalTrials.gov/show/NCT02095132	Wee1 Kinase Inhibitor	*WEE1 Inhibitor AZD1775*	kinase inhibitor	solid	1,2	no
https://ClinicalTrials.gov/show/NCT02091245	XPO1 antagonist	*KPT-330*	other	hemo	1	no
