# Supplementary material for: Importance of Comprehensive Molecular Profiling for Clinical Outcome in Children With Recurrent Cancer
Source: Front Pediatr. 2018 Apr 20;6:114. doi: 10.3389/fped.2018.00114 (PMC5920151; doi:10.3389/fped.2018.00114)
Supplement: Supplementary file 5 [file DataSheet1.DOCX]

**Supplementary material**

Patients

In total, 52 samples were included for complex analysis (Figure 1A; Table S1). There were 3 samples from 2 patients. Sample 8, 17 and 22 were tumor specimens from archived FFPE material (Sample 8) and from fresh tumor biopsies (Samples 17 and 22). Samples 39, 41 and 49 were all cerebrospinal fluid samples for DNA (Sample 39) and RNA (Sample 41) analysis, and for assessment of treatment response (Sample 49). Two samples were received from four patients, i.e. Sample 13 and 15 (primary tumor and its relapse 2 months later), Samples 20 and 21 (different tumors; i.e. lymphoma and glioblastoma, respectively), Samples 7 and 26 (Sample 7 for profiling and Sample 26 for treatment-inclusion screening), and Samples 2 and 28 (primary tumor and sample at progression 5 months later). Samples 22, 26, 41 and 49 were excluded from gender distribution and mean age calculations. Relapses and different tumor sites were considered as individual entities in molecular analysis due to the possible tumor evolution and intratumor heterogeneity.

Methods

Whole exome sequencing (WES)

Genomic and tumor DNA (500 ng) was fragmented to 300 bp using Covaris S2 (Agilent). Adaptor ligation was performed on a Sciclone G3 (Perkin Elmer) using KAPA HTP Library Preparation Kit (Roche). Exomes were enriched with SureSelectXT Clinical Research Exome kit (Agilent) Paired-end sequencing (2x100 bp or 2x150 bp) was performed using either Illumina HiSeq2500 or NextSeq500 to gain an average coverage of 50-100x.Raw sequencing data were processed using CASAVA-1.8.2. Reads were aligned to the human reference genome (hg19/GRCh37) using Biomedical Genomics Workbench (Qiagen) and variant calling was performed above 10% frequency in the tumor DNA. Somatic variants were identified by excluding variants found in blood WES data from the patient, and further analyzed using Ingenuity Variant Analysis (Qiagen). If a germline predisposition was indicated the germline variant calling was performed with a 25% frequency cutoff. The data was analyzed with the same tools as described above for the somatic variants.

RNAsequencing

RNA-sequencing was performed using TruSeq Stranded Total RNA Library Prep Kit and sequenced on a HiSeq2500 or NextSeq500 (Illumina). Paired-end sequencing (2x100 bp or 2x150 bp) was performed to gain an average output of 50-100 M read. FusionMap bioinformatics tool was used for screening of fusion transcripts [11]. If a fusion transcript was identified the sequences was aligned one or both of the involved genes, identifying the fused exons. Sanger Sequencing was performed for validation.

SNP arrays

CytoScan assay (Affymetrix, Santa Clara, USA) was performed on fresh-frozen specimens from tumor biopsies according to the manufacturer´s instructions. OncoScan assay (Affymetrix, Santa Clara, USA) for analysis of FFPE and CSF DNA material was also performed according to the manufacturer´s instructions. OSCHP files from OncoScan and CEL files from the CytoScan assay were imported into NEXUS (BioDiscovery) and used for the analysis and visualization of CNAs and loss of heterozygosity (LOH). CNAs (loss, gain, biallelic loss, or high amplification) and LOH calls for each sample were confirmed by visual inspection and followed by manual interpretation of whole-genome profiles.

Expression array

RNA was reverse-transcribed and used for cRNA synthesis, labelling and hybridization with GeneChip® Human Genome U133 Plus 2.0 Array (Affymetrix) according to the manufacturer’s protocol. The arrays were washed and stained with phycoerytrin conjugated streptavidin using the Affymetrix Fluidics Station 450, and the arrays were scanned in the Affymetrix GeneArray 3000 7G scanner to generate fluorescent images. Cell intensity files (.CEL files) were generated in the GeneChip Command Console Software (AGCC; Affymetrix). The analysis of expression data was performed using Qlucore software (Qlucore AB).
